# Identification of Phytoplasmas Representing Multiple New Genetic Lineages from Phloem-Feeding Leafhoppers Highlights the Diversity of Phytoplasmas and Their Potential Vectors

**DOI:** 10.3390/pathogens10030352

**Published:** 2021-03-16

**Authors:** Wei Wei, Valeria Trivellone, Christopher H. Dietrich, Yan Zhao, Kristi D. Bottner-Parker, Algirdas Ivanauskas

**Affiliations:** 1 Beltsville Agricultural Research Center, Molecular Plant Pathology Laboratory, Agricultural Research Service, United States Department of Agriculture, Beltsville, MD 20705, USA; yan.zhao@usda.gov (Y.Z.); kristi.bottner@usda.gov (K.D.B.-P.); algirdas.ivanauskas@usda.gov (A.I.); 2 Illinois Natural History Survey, Prairie Research Institute, University of Illinois, Champaign, IL 61820, USA; valeria.trivellone@gmail.com (V.T.); chdietri@illinois.edu (C.H.D.)

**Keywords:** genetic lineage, *i*PhyClassifier, insect-borne plant pathogens

## Abstract

Phytoplasmas are obligate transkingdom bacterial parasites that infect a variety of plant species and replicate in phloem-feeding insects in the order Hemiptera, mainly leafhoppers (Cicadellidae). The insect capacity in acquisition, transmission, survival, and host range directly determines the epidemiology of phytoplasmas. However, due to the difficulty of insect sampling and the lack of follow-up transmission trials, the confirmed phytoplasma insect hosts are still limited compared with the identified plant hosts. Recently, quantitative polymerase chain reaction (qPCR)-based quick screening of 227 leafhoppers collected in natural habitats unveiled the presence of previously unknown phytoplasmas in six samples. In the present study, 76 leafhoppers, including the six prescreened positive samples, were further examined to identify and characterize the phytoplasma strains by semi-nested PCR. A total of ten phytoplasma strains were identified in leafhoppers from four countries including South Africa, Kyrgyzstan, Australia, and China. Based on virtual restriction fragment length polymorphism (RFLP) analysis, these ten phytoplasma strains were classified into four distinct ribosomal (16Sr) groups (16SrI, 16SrIII, 16SrXIV, and 16SrXV), representing five new subgroups (16SrI-AO, 16SrXIV-D, 16SrXIV-E, 16SrXIV-F, and 16SrXV-C). The results strongly suggest that the newly identified phytoplasma strains not only represent new genetic subgroup lineages, but also extend previously undiscovered geographical distributions. In addition, ten phytoplasma-harboring leafhoppers belonged to seven known leafhopper species, none of which were previously reported insect vectors of phytoplasmas. The findings from this study provide fresh insight into genetic diversity, geographical distribution, and insect host range of phytoplasmas. Further transmission trials and screening of new potential host plants and weed reservoirs in areas adjacent to collection sites of phytoplasma harboring leafhoppers will contribute to a better understanding of phytoplasma transmission and epidemiology.

## 1. Introduction

Phytoplasmas are a large group of phloem-restricted, cell wall-less bacteria that infect nearly a thousand plant species and cause serious economic loss worldwide. In nature, phytoplasmas are transmitted by phloem sap feeding insect vectors, mainly leafhoppers, in a persistent-propagative manner [1,2,3]. First, phloem feeding insects obtain phytoplasmas from diseased plants during feeding, and then the phytoplasmas penetrate the intestinal wall of the insect (the first barrier) and circulate in the hemolymph. The phytoplasmas further enter the salivary glands (the second barrier) and multiply there. At this point, the insects become vectors. Once they feed on healthy plants, the plants become infected [1,2,3]. The life cycle of phytoplasma involves phytoplasma, plant (healthy and infected), and vector (egg, larva, pupa, and adult stages), which collectively determines the complex relationships and interactions among tri-partite components of phytoplasma pathosystem in a broad ecological context.

Despite numerous efforts, pure phytoplasma culture has not been established in vitro thus far. Like many other unculturable bacteria, higher rank taxa of phytoplasmas (Tenericutes/Mollicutes/Acholeplasmatales/incertae sedis—Family II) were named in the absence of type genus and species, and the *Candidatus* status is used for reserving the putative lower rank taxa (Genus and Species). Phytoplasmas are currently assigned in the provisional genus ‘*Candidatus* Phytoplasma’ [4]. So far, 45 ‘*Candidatus* Phytoplasma’ species have been formally named under this provisional genus based on the 16S rRNA gene sequence [5,6,7,8,9]. Phytoplasmas are phylogenetically coherent and belong to a single clade, but they are also highly diverse. Phytoplasmas have been classified into 36 ribosomal groups, and more than 150 subgroups based on mutually distinct 16S rRNA gene restriction fragment length polymorphism (RFLP) patterns [6,8,10,11,12,13]. In addition to the 16S rRNA gene marker, multi-locus sequence analysis (MLSA) using other genetic markers has also been widely used for finer differentiation of closely related phytoplasmas. These markers include genes encoding ribosomal proteins (*rp*), protein translocase subunit *Sec*Y, and translation elongation factor Tu-EF [8,14]. For example, MLSA characterization based on 16S rRNA, *rp*, and *sec*Y genes revealed azalea little leaf phytoplasmas belonged to a distinct lineage within the aster yellows phytoplasma group [14].

In plants, phytoplasma infection induces various symptoms including virescence (flower petals turning green), phyllody (leafy flowers), cauliflower-like inflorescence, and witches’-broom, thereby altering plant morphology, growth patterns, and architecture [15,16,17,18]. A phytoplasma effector protein, SAP54, identified in aster yellows witches’-broom phytoplasma, can manipulate plant host morphology such as turning normal flowers into leafy and virescent ones, and increase feeding and egg laying of leafhoppers [19]. In insect hosts, phytoplasma can induce host range expansion and host shift of the vector. For instance, the host range of the corn leafhopper, *Dalbulus maidis* expanded after acquiring an aster yellows phytoplasma strain [20]. In addition, host shift of stolbur phytoplasma vector, *Hyalesthes obsoletus* from field bindweed to stinging nettle was observed in northern Italy [21,22]. Due to possible vector-mediated host range expansion and host shift, phytoplasma may “infect” nonspecific plants without showing symptoms. However, most known phytoplasmas have been discovered in symptomatic plants (mainly crops and ornamental plants) in managed agroecosystems and plantation forests with low biodiversity and high inputs. Subsequent insect vector surveys have always been conducted in the same agroecosystems and plantations where the phytoplasmas were found. Compared with the number of known plant hosts, far less phytoplasma insect vectors have been identified because of the difficulty of insect sampling and the lack of follow-up transmission trials. These facts indicate that phytoplasma studies in natural habitats (non-crop and non-plantation areas) and asymptomatic plants have been largely overlooked [23].

Recently, Trivellone et al. [24] used quantitative polymerase chain reaction (qPCR) to screen 227 leafhopper specimens collected worldwide in natural habitats and reported the presence of six phytoplasma strains not yet characterized. The purpose of the present study is to characterize and classify phytoplasmas present in the collection. A total of 76 specimens, including 56 specimens analyzed by qPCR [24] and 20 unexamined new specimens, were subjected to semi-nested PCR, a widely used phytoplasma detection and molecular diagnostic approach. The results showed that a total of ten insect specimens harbored phytoplasmas, nine of which were previously unknown strains. The newly discovered phytoplasma strains represented five new subgroups of four distinct ribosomal (16Sr) groups. In addition, ten phytoplasma-harboring leafhoppers were revealed as phytoplasma insect ”hosts” although they belonged to seven known leafhopper species. The findings from this study provided insight into phytoplasma genetic diversity and the phytoplasma disease pathosystem. The findings will have the guiding significance for in-depth screening of new plant hosts and weed reservoirs of phytoplasmas. Most importantly, the findings strongly suggest that screening leafhoppers is also a very feasible strategy for detecting and predicting potential phytoplasma diseases, especially in the native or cultivated areas of asymptomatic phytoplasma plant hosts.

## 2. Results and Discussion

### 2.1. Phytoplasma Detection in Leafhopper Samples

**Figure 1:**
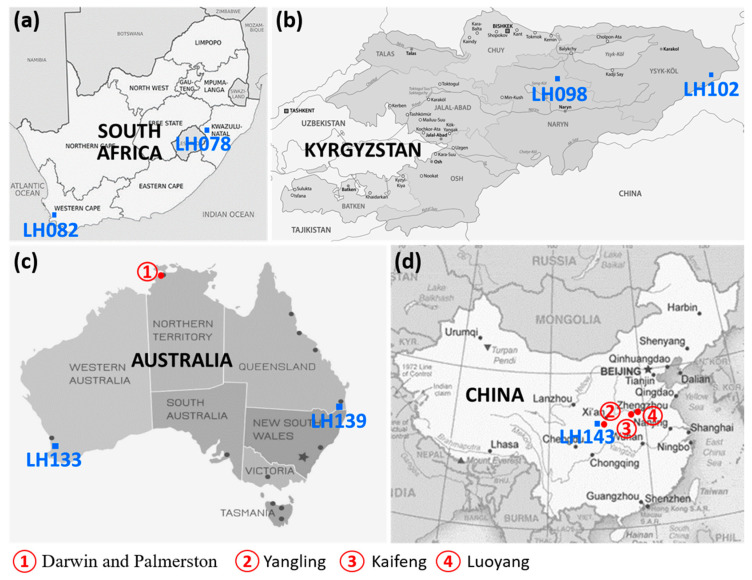
A summary map of seven leafhopper collection sites in four countries where phytoplasmas were detected. (**a**) South Africa; (**b**) Kyrgyzstan; (**c**) Australia, and (**d**) China. Seven sites (blue squares) include LH078, LH082, LH098, LH102, LH133, LH139, and LH143. LH represents LeafHopper, and the number after LH indicates collection event number. Red dots and red numbers 1–4 in the circles indicate where bermudagrass white leaf diseases were found.

The presence of phytoplasma in 76 leafhopper samples collected in natural habitats was investigated by semi-nested PCR amplification employing phytoplasma specific primers. Amplicons of around 1.5 kb were obtained from ten single leafhopper specimens (Table 1, Supplementary Figure S1 and Table S1); among them, six specimens were phytoplasma-positive in qPCR-based prescreening [24]. The ten phytoplasma-positive leafhopper specimens were collected from South Africa, Kyrgyzstan, Australia, and China, respectively (Table 1 and Figure 1, [24]). These leafhoppers belonged to seven leafhopper species, *Leofa dispar, Pravistylus exquadratus, Neoaliturus opacipennis, Macrosteles sordidipennis, Mayawa capitata, Mayawa affinifacialis,* and *Acharis ussuriensis* (Table 1, [24,25]).

### 2.2. Candidatus Phytoplasma’ Species Affiliation of the Leafhopper Harbored Phytoplasmas

PCR products amplified from ten leafhopper samples were sequenced and the obtained sequences covered a nearly full-length 16S rRNA gene and a partial 16S-23S RNA intergenic region. The sequences were deposited to the GenBank (Accession numbers MW281484-MW281493, Table 1). Each phytoplasma strain was given a strain name. For example, the phytoplasma strain identified from LH102-1 leafhopper sample was named as PLH102-1. PLH represents Phytoplasma strain identified in LeafHopper, and numbers are the leafhopper sample codes (please see Materials and Methods). Sequence BLAST search against the *i*PhyClassifier database [13] revealed that strains PLH102-1, PLH098-1, and PLH133-1 were most closely related to the reference strains of ‘*Candidatus* Phytoplasma asteris’, ‘*Candidatus* Phytoplasma pruni’ (*rrn*A and *rrn*B), and ‘*Candidatus* Phytoplasma brasiliense’, respectively. The similarity of 16S rRNA sequence was 98.7%, 99.1% and 97.7%, respectively. Strains PLH078-1, PLH078-12, PLH082-1, PLH082-2, PLH139-1, PLH143-1, and PLH143-5 were closely related to the reference strain of ‘*Candidatus* Phytoplasma cynodontis’, sharing 98.2–98.6% sequence similarity in 16S rRNA gene.

### 2.3. Virtual RFLP Analysis of PLH Phytoplasma Strains

**Figure 2:**
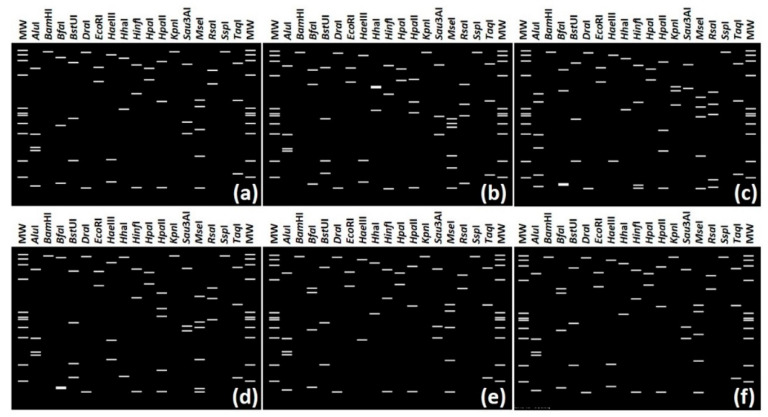
Distinct virtual restriction fragment length polymorphism (RFLP) patterns from in silico digestions of 16S rRNA gene F2nR2 fragments from ten phytoplasma strains. (**a**) PLH078-1, PLH078-12, PLH082-1, PLH082-2, (**b**) PLH098-1, (**c**) PLH102-1, (**d**) PLH133-1, (**e**) PLH139-1, (**f**) PLH143-1, and PLH143-5. Recognition sites for the following 17 restriction enzymes were used in the simulated digestions: *Alu*I, *Bam*HI, *Bfa*I, *Bst*UI (*Tha*I), *Dra*I, *Eco*RI, *Hae*III, *Hha*I, *Hinf*I, *Hpa*I, *Hpa*II, *Kpn*I, *Sau*3AI (*Mbo*I), *Mse*I, *Rsa*I, *Ssp*I, and *Taq*I. MW, *ϕ*X174 DNA-*Hae*III digestion as a marker.

**Figure 3:**
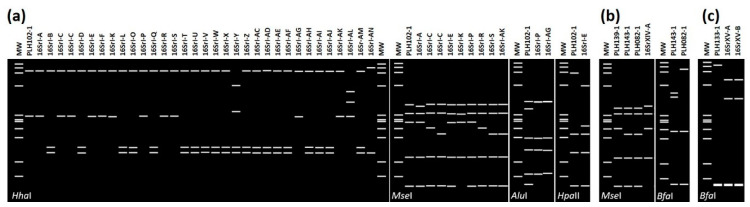
Identification of five new subgroups belonging to different phytoplasma classification groups based on the in silico RFLP patterns derived from key restriction enzyme digestions. (**a**) The new subgroup pattern 16SrI-AO (PLH102-1) can be differentiated from the other 33 subgroup patterns of the 16rI group by *Hha*I, *Mse*I, *Alu*I, and *Hpa*II enzyme digestions (first separated from 23 subgroups, and then from 7, 2, and 1 16SrI subgroups, respectively). (**b**) Three new subgroup patterns 16SrXIV-D (PLH139-1), 16SrXIV-E (PLH143-1 and PLH143-5), and 16SrXIV-F (PLH078-1, PLH078-2, PLH082-1, and PLH082-2) can be differentiated from previously reported 16SrXIV-A subgroup by *Mse*I digestion alone. 16SrXIV-E and 16SrXIV-F can be separated by *Bfa*I digestion. Subgroups 16SrXIV-B and 16SrXIV-C were abolished in this study due to same RFLP pattern with that of 16SrXIV-A (details see Table 3). (**c**) Distinction of new subgroup pattern 16SrXV-C (PLH133-1) from previously reported 16SrXV-A and 16SrXV-B subgroup patterns can be accomplished by *Bfa*I digestion alone. MW, *ϕ*X174 DNA-*Hae*III digestion as a marker.

The virtual RFLP analysis of F2nR2 (1.25 kb) 16S rRNA gene fragment was conducted by *i*PhyClassifier [13], and six mutually distinct RFLP patterns were identified from ten PLH strains (Figure 2). The RFLP profiles of strains PLH078-1, PLH078-12, PLH082-1, and PLH082-2 were identical (Figure 2a), and strains PLH143-1 and PLH143-5 also had the same RFLP pattern (Figure 2f). Strains PLH098-1, PLH102-1, PLH133-1, and PLH139-1 had distinct RFLP patterns (Figure 2b–e). These ten phytoplasma strains belonged to four different phytoplasma classification groups including (i) Aster yellow (16SrI) group, (ii) X-disease (16SrIII) group, (iii) Bermudagrass white leaf (16SrXIV) group, and (iv) Hibiscus witches’-broom (16SrXV) group. The phytoplasmas were further classified into six subgroups as detailed below; five of them represented new subgroups (Table 1 and Figure 3).

Strain PLH102-1 belonged to 16SrI group and exhibited a collective RFLP profile different from those of all 33 previously established subgroups in the group 16SrI. As shown in Figure 3a, virtual digestion with restriction enzyme *Hha*I alone was able to distinguish strain PLH102-1 from 23 previously established 16SrI subgroups. Virtual RFLP patterns from *Mse*I, *Alu*I, and *Hpa*II digestions separated strain PLH102-1 from the remaining ten 16SrI subgroups. Similarity coefficients derived from virtual RFLP analysis of 16S rRNA genes of strain PLH102-1 and other 16SrI subgroups were less than or equal to 0.97, the threshold for a new subgroup delineation [26]. Therefore, strain PLH102-1 was designated as a new subgroup 16SrI-AO (Table 2 [10,27,28,29,30,31,32,33,34,35,36,37,38,39,40,41,42,43,44,45,46,47,48,49,50], some of previously reported 16SrI subgroups were reassigned due to duplication).

Strains PLH078-1, PLH078-12, PLH082-1, PLH082-2, PLH139-1, PLH143-1, and PLH143-5 (Table 1) were classified into bermudagrass white leaf (16SrXIV) group, representing three new subgroups. So far, three 16SrXIV subgroups (16SrXIV-A, 16SrXIV-B, and 16SrXIV-C) have been reported [51,52,53]. However, RFLP profiles of 16SrXIV-A, 16SrXIV-B, and 16SrXIV-C are identical and similarity coefficient between them is 1.00 (Table 3). This means they are same subgroup (16SrXIV-B and 16SrXIV-C are also 16SrXIV-A). Therefore, 16SrXIV-B, and 16SrXIV-C were abolished in this study (Table 3). Similarity coefficients between the representative strains of each of the three new 16SrXIV subgroups and the representative strain of 16SrXIV-A were 0.81-0.90 (Table 3). The scores were less than or equal to 0.97, which is the threshold for a new subgroup delineation [26]. The three new 16SrXIV subgroups were designated as 16SrXIV-D, 16SrXIV-E, and 16SrXIV-F. Strain PLH139-1, PLH143-5, and PLH082-1 represent three new subgroups, respectively. They can be differentiated from previously reported 16SrXIV-A subgroup by *Mse*I digestion alone, and 16SrXIV-E and 16SrXIV-F can be separated by *Bfa*I digestion (Figure 3b).

### 2.4. Phylogenetic Positions of Newly Identified PLH Strains

Strain PLH133-1 belonged to Hibiscus witches’-broom (16SrXV) group and represented a new subgroup 16SrXV-C. 16SrXV-C and previously reported 16SrXV-A and 16SrXV-B subgroups can be differentiated by *Bfa*I digestion alone (Figure 3c).

Strain PLH098-1 exhibited a collective virtual RFLP profile identical to that of subgroup J of the X-disease (16SrIII) group (Figure 2b). Therefore, PLH098-1 is a new member of the subgroup, 16SrIII-J.

**Figure 4:**
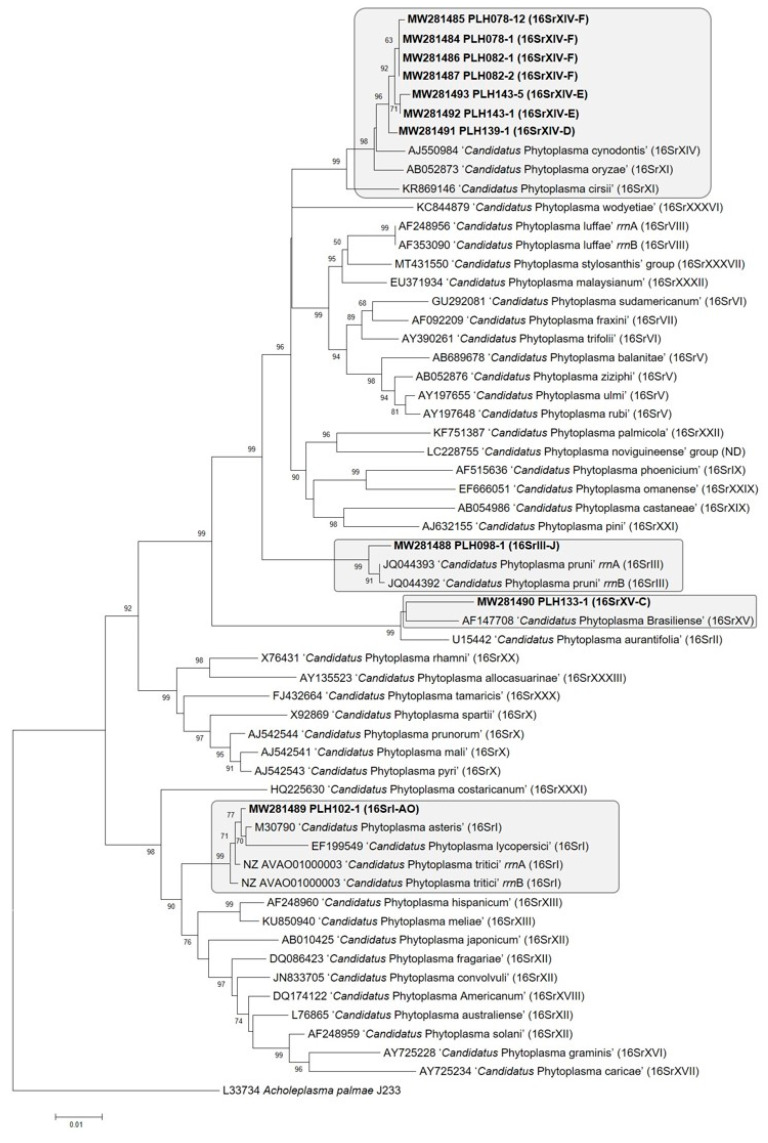
A phylogenetic tree inferred from minimum evolution (ME) analysis of 16S rRNA gene sequences. The evolutionary distances were computed using the maximum composite likelihood method implemented in the software package MEGA-6 [54]. The ME tree was searched using the Close Neighbor Interchange (CNI) algorithm at a search level of 3. The neighbor-joining algorithm was used to generate the initial tree. The reliability of the analysis was subjected to a bootstrap test with 1000 replicates. The percentage values of replicate trees in which the associated taxa clustered together in the bootstrap test are shown next to the branches. The taxa used in the phylogenetic tree construction included reference strains of 44 previously described ‘*Candidatus* Phytoplasma’ species (The sequence of one new ‘*Candidatus* Phytoplasma’ species has not been released yet); phytoplasma strains identified in the current study are highlighted in bold. *Acholeplasma palmae* served as an out-group during the phylogenetic tree construction. The scale bar represents evolutionary distance in nucleotide substitutions per base position. Ten newly identified phytoplasma strains fell in four distinct subclades, highlighted in gray boxes.

The relationships between individual PLH strains and reference strains of previously described ‘*Candidatus* Phytoplasma’ species were examined by phylogenetic analysis of 16S rRNA gene sequences using Mega 6 [54]. The tree topology demonstrated that the ten PLH strains belonged to the monophyletic phytoplasma clade and were phylogenetically related to four different subclades (Figure 4). Strain PLH102-1 clustered with reference strains of ‘*Candidatus* Phytoplasma asteris’, ‘*Candidatus* Phytoplasma lycopersici’, and ‘*Candidatus* Phytoplasma tritici’ (*rrn*A and *rrn*B), which are classified in 16SrI group. Strain PLH098-1 and ‘*Candidatus* Phytoplasma pruni’ (*rrn*A and *rrn*B) reference strains grouped together and fell into 16SrIII group (Figure 4).

Strains PLH078-1, PLH078-12, PLH082-1, PLH082-2, PLH139-1, PLH143-1, and PLH143-5 were most closely related to ‘*Candidatus* Phytoplasma cynodontis’ reference strain (16SrXIV group), and then ‘*Candidatus* Phytoplasma oryzae’ reference strain (16SrXI group), and ‘*Candidatus* Phytoplasma cirsii’ reference strain (16SrXI group). In the process of updating the phytoplasma classification, it was difficult to determine the classification of some phytoplasma strains that are related to the groups 16SrXI and 16SrXIV. This is because the similarity coefficients between two groups of strains are not always less than 0.85 (the threshold for a new group, [12]). We propose to combine these two groups into one group [55].

Strain PLH133-1 was closely related to the ‘*Candidatus* Phytoplasma brasiliense’ reference strain, which belongs to 16SrXV group. The result from the phylogenetic analysis was consistent with that of sequence BLAST and virtual RFLP analyses.

### 2.5. The Geographical Distribution and Potential Vector Relationship between the Newly Identified PLH Strains and the Known Phytoplasma Strains

PLH phytoplasma strains were found in leafhoppers collected from four different countries. The individual sampling locations were marked in Figure 1 based on the coordinates (Table 1), and the distance between two locations was calculated by Latitude/Longitude Distance Calculator, respectively.

#### 2.5.1. Kyrgyzstan

Two Kyrgyzstan strains (PLH098-1 and PLH102-1) were detected in two different leafhopper species, *Neoaliturus opacipennis,* and *Macrosteles sordidipennis*, which were collected from Jct. Kokerim and Kugart Rivers, and Ak-Shyrrak (244 km apart, Table 1 and Figure 1b). These two phytoplasma strains belonged to two different subgroups in two distinct groups (a new subgroup, 16SrI-AO, and an existing subgroup 16SrIII-J, Table 1 and Table 2, Figure 2c and Figure 3a). Neither group 16SrI nor group 16SrIII phytoplasma has been reported in Kyrgyzstan. In fact, the only phytoplasma disease previously reported in the country was potato Stolbur disease [56]. In Europe, the same potato disease was known to be associated with a group 16SrXII phytoplasma.

#### 2.5.2. China

Two strains, PLH143-1 and PLH143-5 were detected in the leafhopper species, *Acharis ussuriensis*, in Zhouzhi county, Shaanxi, China (Table 1; Figure 1d). The virtual RFLP patterns of the two strains were identical (Figure 2f), and they represented a new subgroup 16SrXIV-E (Figure 3b). To date, more than ten different groups (16SrI, 16SrII, 16SrIII, 16SrV, 16SrVI, 16SrXI, 16SrXII, 16SrXIV, 16SrXIX, 16SrXXI, 16SrXXX, and 16SrXXXII) of phytoplasma strains have been found in China [57,58]. Interestingly, a ‘*Candidatus* Phytoplasma cynodontis’-related phytoplasma strain (EU999999, a 16SrXIV-A variant, unpublished) which induces white leaf disease in bermudagrass (*Cynodon dactylon*) was found in Yangling, Shaanxi, China (Figure 1d). Zhouzhi County and Yangling are about 25 km apart. Namely, the two PLH143 strains and the bermudagrass white leaf phytoplasma (BGWL) Yangling strain were discovered in the same geographical area. In addition, BGWL strains were also found in Kaifeng and Luoyang, Henan, China [59], 600 km away from Zhouzhi county (Figure 1d). These facts increased the possibility of *Acharis ussuriensis* as a potential insect vector of bermudagrass white leaf disease in Shaanxi and Henan provinces, China.

#### 2.5.3. Australia

Genetic diversity of Australian phytoplasma strains is also very significant and known strains have been classified into many existing 16Sr groups (16SrI, 16SrII, 16SrIII, 16SrX, 16SrXI, 16SrXII, 16SrXIV, 16SrXXIII, 16SrXXV, and 16SrXXIX [57,58]). Among them, there are many economically important strains such as Australian grapevine yellows phytoplasma, strawberry lethal yellowing phytoplasma, and tomato big bud phytoplasma. One Australian strain PLH133-1, which was identified in *Mayawa capitata* represented a new subgroup 16SrXV-C (Table 1; Figure 3c) and shared 97.7% similarity in 16S rRNA gene sequence with that of ‘*Candidatus* Phytoplasma brasiliense’. This is the first report of a 16SrXV phytoplasma strain in Australia.

The other Australian strain PLH139-1 was found in *Mayawa affinifacialis* [24], which was collected in Flagstone Creek Protection Park, Eastern Queensland (Figure 1c and Table 1). It shared 98.6% similarity with that of the ‘*Candidatus* Phytoplasma cynodontis’ reference strain (16SrXIV-A) and represented a new subgroup 16SrXIV-D (Figure 3b; Table 3). Interestingly, strain PLH139-1 shared 98.5% similarity in 16S rRNA gene sequence with a known Australian BGWL strain (AF509321, [60]). The BGWL strain was discovered during field surveys of sugarcane in the Darwin and Palmerston regions of Australia’s Northern Territory (Figure 1c). The insect vector that transmits Australian BGWL strain has not been identified, and *Mayawa affinifacialis* should be a good candidate for further verification of transmission trials.

#### 2.5.4. South Africa

Four South African strains (PLH078-1, PLH078-12, PLH082-1, and PLH082-2) were detected in leafhoppers collected from Arthurs Seat Hill, KwaZulu-Natal Province, and Wemmershoek Dam, North of Franschhoek in Western Cape province (Figure 1a and Table 1). Although the two sampling sites are 1120 km apart, the four strains shared 98.2-98.5% 16S rRNA gene sequence similarity with that of the ‘*Candidatus* Phytoplasma cynodontis’ reference strain (16SrXIV-A). They all belonged to a new subgroup 16SrXIV-F (Figure 3b; Table 3). These phytoplasma strains were found in two different leafhopper species, *Leofa dispar*, and *Pravistylus exquadraus*. Neither of these leafhopper species were previously identified as phytoplasma insect vectors.

Phytoplasma disease is rarely reported in South Africa. Prior to this study, only two reports had been published. One reported a mixed infection of 16SrII and 16SrXII groups of phytoplasmas in grapevine [61] (sequences are not available in NCBI database). The other one reported aster yellows phytoplasma strain SA-Vdal (GQ365729, [62]), which belongs to 16SrI group and also infects grapevine. In addition, there is one more unpublished strain, sugarcane yellows phytoplasma type I strain ScYP I-Afr (AF056095) in 16SrIII group. Different from China and Australia, no BGWL phytoplasma has been reported in South Africa although bermudagrass is native to savannas of Africa. In conclusion, this is the first time that ‘*Candidatus* Phytoplasma cynodontis’-related phytoplasma strains have been found in South Africa.

### 2.6. An Overview of Widespread BGWL Phytoplasma Disease and Its Possibility in South Africa

Among ten newly identified PLH phytoplasma strains, seven strains were within Bermudagrass white leaf (16SrXIV) group and represented three distinct genetic subgroup lineages (16SrXIV-D, 16SrXIV-E, and 16SrXIV-F). Bermudagrass is a fast-growing grass that is spread by seeds and its vegetative propagules, stolons, and rhizomes, and quickly colonize new areas where it grows into dense mats [63,64]. As such, it is one of the most widely used turf grasses for sports fields, golf courses, and general use lawns in tropical and subtropical areas [63,64]. It is therefore not surprising that BGWL disease is widespread in many countries on different continents including (1) Asia: Singapore [65], Iran [52], Turkey [66], Thailand [67], India [68,69,70], Malaysia [71,72], Myanmar [73], Saudi Arabia [74], China [59], and Vietnam (unpublished); (2) Africa: Sudan [75], Kenya, Tanzania and Uganda [76], and Ethiopia (unpublished); (3) Europe: Italy [77], Serbia and Albania [53]; (4) North America: Cuba [78]; and (5) Australia: Australia [79]. All BGWL strains belong to 16SrXIV group with minor genetic variations except for an Ethiopian strain (DQ305983) and a Cuban strain (AY742327). They are classified into 16SrIII and 16SrXVI group, respectively. In South Africa, the potential risk of BGWL disease should not be underestimated because bermudagrass is native to Africa. In addition, ‘*Candidatus* Phytoplasma cynodontis’-related BGWL strains (16SrXIV group) have been reported in several African countries, such as Sudan [75], Kenya, Tanzania, and Uganda [76]. Identification of ‘*Candidatus* Phytoplasma cynodontis’-related phytoplasmas (16SrXIV-F) in leafhopper samples collected at two geographical locations in South Africa (Figure 2a) further indicate the presence of such risk.

On the other hand, bermudagrass is also considered as one of the most “troublesome” agricultural and environmental invasive weeds in the world. This is because bermudagrass is drought tolerant, flood resistant and can regenerate quickly after fire, and compete for space and nutrients with many crops, especially, sugarcane (*Saccharum officinarum*, [80]). Sugarcane, a large perennial grass species in the genus *Saccharum*, is one of the important sources of raw materials for sugar and ethanol production [81]. Bermudagrass has been recognized as a host for many sugarcane diseases, such as sugarcane ratoon stunting disease [82], and sugarcane mosaic disease [83]. In addition, BGWL phytoplasma strains are also very closely associated with strains that cause sugarcane white leaf (SCWL, 16SrXI-B), sugarcane grassy shoot (SCGS) diseases (16SrXI-B). The studies on BGWL, SCWL, and SCGS diseases have always been inseparable [51,59,77,79]. In this study, five leafhoppers that harbor ‘*Candidatus* Phytoplasma cynodontis’-related strains were found in South Africa, Australia, and China. Considering that BGWL disease has already occurred in Australia and China, it is likely to occur in South Africa. The bermudagrass and sugarcanes in the vicinity of the collection sites where the leafhoppers tested positive for BGWL phytoplasma strains should be investigated as a priority in future studies.

### 2.7. An Expansion of Potential Insect Host Range and Phytoplasma Genetic Diversity

Although phytoplasma-harboring leafhoppers examined in the present study are not new species, none of the them were unveiled as phytoplasma “hosts”. It is worth mentioning that phytoplasma detection in the present study was based on the DNA extraction from the dissected abdomen of a single insect specimen, instead of pooled insect specimens commonly adopted to screen insect vectors. Nine of the ten phytoplasma-positive leafhoppers could be detected by direct PCR (the first step of two-step semi-nested PCR, Supplemental Figure S1). This indicated that phytoplasma titers in the leafhopper body were relatively high; that is, these leafhopper species are likely to be phytoplasma insect vectors, which needs to be confirmed by further transmission trials.

Phytoplasmas are believed to be highly diverse. This is mainly due to their complex life cycle of living in two different hosts, plants, and insects as well as their long evolutionary history [84]. In this study, the ten PLH strains were identified in 76 leafhoppers collected from previously overlooked natural habitats. Nine of them (90%) represent five new subgroups and one of them (10%) is a known subgroup (Table 1, Table 2 and Table 3; Figure 1, Figure 2 and Figure 3). The results strongly indicate that these phytoplasmas represent multiple new subgroup lineages, which broadens our understanding in phytoplasma genetic diversity, especially those of insect host origin.

In summary, the new PLH strains identified in this study either represent a new genetic subgroup lineage or mark an extension of the geographic distribution of known phytoplasmas. All PLH-harboring leafhopper species are new insect “hosts” of phytoplasmas. The findings provide insight into the genetic diversity, geographical distribution, and potential insect host range of phytoplasmas. Further transmission trials and screening of new potential host plants and weed reservoirs in the vicinity of phytoplasma-harboring leafhoppers will help us to gain a better understanding of phytoplasma transmission and epidemiology. The findings from this study also support the possibility of screening insect “hosts “of phytoplasmas as a viable strategy for discovery of potential phytoplasma diseases.

## 3. Materials and Methods

### 3.1. Leafhopper Samples and DNA Templates

A total of 76 leafhopper specimens collected in natural habitats were used in this study (Supplemental Table S1). Among the 76 specimens, 56 of them had previously been screened for the presence of phytoplasmas by qPCR and six tested positive [24]; the remaining 20 were previously untested leafhopper specimens from the same collection sites where six phytoplasma-positive leafhoppers were found. The leafhopper DNA was extracted from single insect specimens by employing a non-destructive method described by Trivellone et al. [24] The dissected abdomen of each specimen was incubated overnight in 400 µL 1X TES buffer (20 mM Tris, 10 mM EDTA, 0.5% SDS, pH 7.8) and 4 µL Proteinase K (20 mg/µL) at 56 °C. After incubation, the abdomen was removed, and the remaining solution was transferred to a new tube. The same volume of chloroform (400 µL) was added to the tube, vortexed, and centrifuged at 11,000 rpm for 10 min at 4 °C. The upper aqueous layer is transferred to a new tube and chloroform extracted again. The upper aqueous layer was transferred to another new tube, and 400 µL of ice-cold isopropanol was added, vortexed and centrifuged for 15 min at 4 °C at 12,000 rpm. The supernatant was discarded, and the pellet was washed twice with 500 µL of ice-cold 96% ethanol and centrifuged again. The DNA pellet was dried for 20 min and re-suspended in 50 µL of TE buffer (pH 7.8). The DNA was used for semi-nested PCR amplification. Each collected leafhopper sample was coded, for example, LH078-1, LH represents LeafHopper, and numbers before and after hyphen (-) indicate collection event number, and randomly selected insect specimen number during the same collection. According to the coordinates of the sampling sites, the distance between different sites was calculated by Latitude/Longitude Distance Calculator (https://www.nhc.noaa.gov/gccalc.shtml) accessed on 29 November 2019.

### 3.2. PCR Detection and Sequencing of PCR Products

Detection of phytoplasmas was performed by semi-nested PCR amplification of 16S rRNA gene using universal primer pair P1/16S-SR (Direct PCR, the first step of semi-nested PCR, P1: 5′-AAGAGTTTGATCCTGGCTCAGGATT-3′/16S-SR: 5′-GGTCTGTCAAAACTGAAGATG-3′, [85]) followed by P1A/16S-SR (semi-nested PCR, P1A: 5′-AACGCTGGCGGCGCGCCTAATAC-3′, [86]). Each PCR reaction mixture (25 μL) contains 1 μL extracted insect DNA, 200 μM of each dNTP, 4 μM of each primer, 15 mM MgCl_2_, 2.5 units of LATaq DNA polymerase (Takara Bio USA, Madison, WI). PCRs were operated for 38 cycles and the following conditions were used: denaturation at 94 °C for 1 min, annealing at 55 °C for 2 min and extension at 72 °C for 3 min (10 min in the final extension). Diluted PCR product (1:20) from the first amplification was used as template in the semi-nested PCR. Amplicons were analyzed by electrophoresis through an agarose gel, incorporated with SYBR Safe DNA Gel Stain (Invitrogen, Carlsbad, CA, USA) and visualized with a UV transilluminator. PCR amplified products were cleaned by gel extraction using the QIAquick Gel Extraction Kit (Qiagen, Germantown, MD, USA) and sequenced with an automated DNA sequencer (Macrogen Inc., Rockville, MD USA). Raw sequence assembly and analysis was conducted by Seqman, an application of DNAStar LaserGene software package (DNAStar, Madison, WI). All sequences were deposited into NCBI nucleotide database (Accession numbers MW281484-MW281493, Table 1).

### 3.3. Virtual RFLP Analysis and Phylogenetic Analysis

In order to further identify and classify the phytoplasma strains, computer simulated virtual RFLP analysis and similarity coefficient calculations were performed for the obtained 16S rRNA gene sequences by using the online tool, *i*PhyClassifier [13]. Digestions of 17 key restriction enzymes (*Alu*I, *Bam*HI, *Bfa*I, *Bst*UI (*Tha*I), *Dra*I, *Eco*RI, *Hae*III, *Hha*I, *Hinf*I, *Hpa*I, *Hpa*II, *Kpn*I, *Sau*3AI (*Mbo*I), *Mse*I, *Rsa*I, *Ssp*I, and *Taq*I) were simulated. A phylogenetic tree was constructed using the Minimum Evolution (ME) method implemented in the software package MEGA-6 [54]. The ME tree was searched using the Close Neighbour Interchange (CNI) algorithm at a search level of 2. The neighbor-joining algorithm was used to generate the initial tree. The reliability of the analysis was subjected to a bootstrap test with 1000 replicates. The percentage values of replicate trees in which the associated taxa clustered together in the bootstrap test are shown next to the branches. The taxa used in the phylogenetic tree construction included reference strains of all previously described ‘*Candidatus* Phytoplasma’ species except for a new *Candidatus* species because the sequence has not been released yet. *Acholeplasma palmae* served as an out-group during the phylogenetic tree construction.

## Figures and Tables

**Table 1:** Identification and classification of phytoplasmas detected in leafhopper samples collected from natural habitats.

Phytoplasma Strain *	Leafhopper Species	GenBank Accession Number (This Study)	Phytoplasma 16Sr Group /Subgroup Classification	Country	GenBank Accession Number (Trivellone et al. [24])	Coordinate X/ Coordinate Y
PLH078-1	*Leofa dispar*	MW281484	16SrXIV-F	South Africa	MW473669	28°53′59.1″ S/ 29°26′05.0″ E
PLH078-12	*Leofa dispar*	MW281485	16SrXIV-F	South Africa	not analyzed	28°53′59.1″ S/ 29°26′05.0″ E
PLH082-1	*Pravistylus exquadratus*	MW281486	16SrXIV-F	South Africa	MW473673	33°51′01.7″ S/ 19°03′16.3″ E
PLH082-2	*Pravistylus exquadratus*	MW281487	16SrXIV-F	South Africa	not analyzed	33°51’01.7″ S/ 19°03′16.3″ E
PLH098-1	*Neoaliturus opacipennis*	MW281488	16SrIII-J	Kyrgyzstan	not sequenced	41°59′11.0″ N/ 75°43′08.0″ E
PLH102-1	*Macrosteles sordidipennis*	MW281489	16SrI-AO	Kyrgyzstan	MW473674	41°47′52.0″ N/ 78°39′44.0″ E
PLH133-1	*Mayawa capitata*	MW281490	16SrXV-C	Australia	MW473671	32°57′20.8″S/ 115°54′40.5″ E
PLH139-1	*Mayawa affinifacialis*	MW281491	16SrXIV-D	Australia	MW473672	27°56′03.4″ S/ 153°04′42.6″ E
PLH143-1	*Acharis ussuriensis*	MW281492	16SrXIV-E	China	MW473670	33°58′52.8″ N/ 108°09′49.8″ E
PLH143-5	*Acharis ussuriensis*	MW281493	16SrXIV-E	China	not analyzed	33°58′52.8″ N/ 108°09′49.8″ E

* PLH represents Phytoplasma strain identified in LeafHopper, and numbers before and after hyphen (-) indicate collection event number, and randomly selected insect specimen number during the same collection, respectively.

**Table 2:** Similarity coefficients derived from virtual RFLP analysis of 16S rRNA genes of PLH102-1 (new subgroup 16SrI-AO) and other 16SrI subgroups.

Strain	GenBank Accession (16SrI Subgroup)	Similarity Coefficient between PHL102-1 and Known 16SrI Subgroup	References
**PLH102-1, Kyrgyzstan**	**MW281489 (16SrI-AO)**	**1.00**	**this study**
AYWB, aster yellows witches’-broom, USA	CP000061 (16SrI-A)	0.95	[10,25]
OYM, onion yellows mild, Japan	AP006628 (16SrI-B)	0.97	[10,26]
CPh, clover phyllody, Canada	AF222065 (*rrn*A, 16SrI-C) ^(a)^	0.96	[10]
CPh, clover phyllody, Canada	AF222066 (*rrn*B, 16SrI-C) ^(a)^	0.95	[10]
PaWB, paulownia witches’-broom, Taiwan	AY265206 (16SrI-D)	0.94	[27]
BBS3, blueberry stunt, USA	AY265213 (16SrI-E)	0.96	[27]
ACLR-AY, apricot chlorotic leaf roll, Spain	AY265211 (16SrI-F)	0.91	[27]
STRAWB2, strawberry multiplier, USA	U96616 (16SrI-K)	0.89	[28]
OnP2, onion proliferation, Lithuania	GU223209 (16SrI-L)	0.94	[29]
98UW166B, aster yellows, USA	AF268405 (16SrI-O)	0.83	[30]
AYIP, aster yellows, Croatia	AF503568 (16SrI-P)	0.97	[31]
CherLL, cherry little leaf, Lithuania	AY034089 (16SrI-Q)	0.89	[32]
ChBL, cherry bunchy leaf, Lithuania	HM067754 (16SrI-R)	0.96	[33]
LcLL, lilac little leaf, Lithuania	HM067755 (16SrI-S)	0.95	[33]
AzLL, azalea little leaf, China	HQ285917 (16SrI-T)	0.89	[34]
PPT-JAL6, potato purple top, Mexico	FJ914650 (16SrI-U)	0.90	[35]
PPT-SON18, potato purple top, Mexico	FJ914642 (16SrI-V)	0.90	[35]
SoySTp-1, Soybean stunt, Cuba	KJ413093 (16SrI-W)	0.94	[36]
BTS, Papaya bunchy top, Cuba	JF781308 (16SrI-X)	0.92	[37]
SoySTp-2, Soybean stunt, Cuba	KJ413094 (16SrI-Y)	0.91	[36]
PBBB, Potato Brotes big bud, Bolivia	AY725209 (16SrI-Z)	0.89	[38,39]
*Fraxinus uhdei* witches’-broom, Colombia	JQ730859 (16SrI-AC)	0.92	[39,40]
BLL, basil little leaf, Cuba	DQ286577 (16SrI-AD)	0.91	[39,41]
Broad bean phytoplasma, Cuba	DQ286953 (16SrI-AE)	0.94	[39,42]
MgPh, Marigold phyllody, Mexico	AY249247 (16SrI-AF)	0.91	[39,43]
NS1P1cB, BS, blueberry stunt, Canada	MH279522 (16SrI-AG) ^(b)^	0.88	[44]
PWWB, Purple woodnettle witches’-broom, Taiwan	KF923395 (16SrI-AH)	0.94	[45]
SFDP, sunflower fasciation, China	JX035903 (16SrI-AI)	0.87	[46]
Bidens-Cba, *’Bidens subalternans’* phytoplasma, Argentina	MH497011 (16SrI-AJ)	0.91	[47]
CgWB1, *Campsis grandiflora* witches’-broom, China	MT106667 (16SrI-AK)	0.90	[48]
CgWB2, *Campsis grandiflora* witches’-broom, China	MT106668 (16SrI-AL)	0.90	[48]
LoofWB-1U4, loofah witches’-broom, Mexico	MN807428 (16SrI-AM) ^(c)^	0.88	[49]
LoofWB-21J9, loofah witches’-broom, Mexico	MN807432 (16SrI-AN) ^(d)^	0.90	[49]

^(**a**)^ GenBank submission in 2001 by Dally, E.L.,Bottner, K.D. and Davis, R.E. ^(**b**)^ This is a re-designation of a subgroup pattern (16SrI-AI) established by Perez-Lopez et al. [40]. The reason for the re-designation is that the subgroup letter 16SrI-AI had been previously published by Zhang et al. [47]. ^(**c**)^ This is re-designation of the subgroup 16SrI-AG reported by Santos-Cervantes et al. [36] as the subgroup letter 16SrI-AG (MH279522) had already been assigned to NS1P1cB, BS, blueberry stunt, Canada. ^(**d**)^ This is re-designation of the subgroup 16SrI-H reported by Santos-Cervantes et al. [36] as the subgroup letter 16SrI-H (KF923395) had already been published by Tseng et al. [46].

**Table 3:** Similarity coefficients derived from virtual RFLP analysis of 16S rRNA genes of 16SrXIV subgroups.

Phytoplasma		Country	GenBank Accession	Subgroup	Reference	1	2	3	4	5	6	7	8	9	10	11
**1**	*‘Candidatus* Phytoplasma cynodontis’	Italy	AJ550984	16SrXIV-A	[50]	1.00										
**2**	**PLH139-1**	Australia	MW281491	16SrXIV-D	**this study**	0.90	1.00									
**3**	**PLH143-1**	China	MW281492	16SrXIV-E	**this study**	0.84	0.94	1.00								
**4**	**PLH082-1**	South Africa	MW281486	16SrXIV-F	**this study**	0.81	0.91	0.97	1.00							
**5**	Bermuda grass white leaf phytoplasma Juyom	Iran	EF444486	16SrXIV-B * (abolished)	[51]	1.00	0.91	0.86	0.82	1.00						
**6**	Bermuda grass white leaf phytoplasma Firoozabad	Iran	EF444485	16SrXIV-B * (abolished)	[51]	1.00	0.90	0.84	0.81	1.00	1.00					
**7**	*‘Candidatus* Phytoplasma cynodontis’ strain 306/13	Serbia	KJ000021	16SrXIV-C * (abolished)	[52]	1.00	0.90	0.84	0.81	1.00	1.00	1.00				
**8**	*‘Candidatus* Phytoplasma cynodontis’ strain 123/13	Serbia	KJ000024	16SrXIV-C * (abolished)	[52]	1.00	0.90	0.84	0.81	1.00	1.00	1.00	1.00			
**9**	*‘Candidatus* Phytoplasma cynodontis’ strain 304/13	Serbia	KP019339	16SrXIV-C * (abolished)	[52]	1.00	0.90	0.84	0.81	1.00	1.00	1.00	1.00	1.00		
**10**	*‘Candidatus* Phytoplasma cynodontis’ strain 305/13	Serbia	KP019340	16SrXIV-C * (abolished)	[52]	1.00	0.90	0.84	0.81	1.00	1.00	1.00	1.00	1.00	1.00	
**11**	*‘Candidatus* Phytoplasma cynodontis’ strain 59/11	Serbia	KF383981	16SrXIV-C * (abolished)	[52]	1.00	0.90	0.84	0.81	1.00	1.00	1.00	1.00	1.00	1.00	1.00

* Subgroups 16SrXIV-B and 16SrXIV-C were abolished because similarity coefficient between 16SrXIV-B, 16SrXIV -C and previously named 16SrXIV-A is 1.00 (underscored), which means 16SrXIV-B and 16SrXIV-C are also 16SrXIV-A.

## Supplementary Materials

The following are available online at https://www.mdpi.com/2076-0817/10/3/352/s1, Supplementary Figure S1: Phytoplasma detection in ten leafhoppers by semi-nested polymerase chain reaction (PCR) amplification of 16S rRNA gene with universal primer pair P1/16S-SR (Direct PCR), followed by amplification with primers P1A/16S-SR (Seminested PCR); Supplementary Table S1: Detection of phytoplasmas in 76 leafhopper samples by semi-nested polymerase chain reaction (PCR), including 56 quantatitive PCR (qPCR) screend and 20 untested leafhopper samples.
