# Supplementary material for: Identification of Phytoplasmas Representing Multiple New Genetic Lineages from Phloem-Feeding Leafhoppers Highlights the Diversity of Phytoplasmas and Their Potential Vectors
Source: Pathogens. 2021 Mar 16;10(3):352. doi: 10.3390/pathogens10030352 (PMC8002289; doi:10.3390/pathogens10030352)
Supplement: Supplementary file 1 [file pathogens-10-00352-s001.pdf]

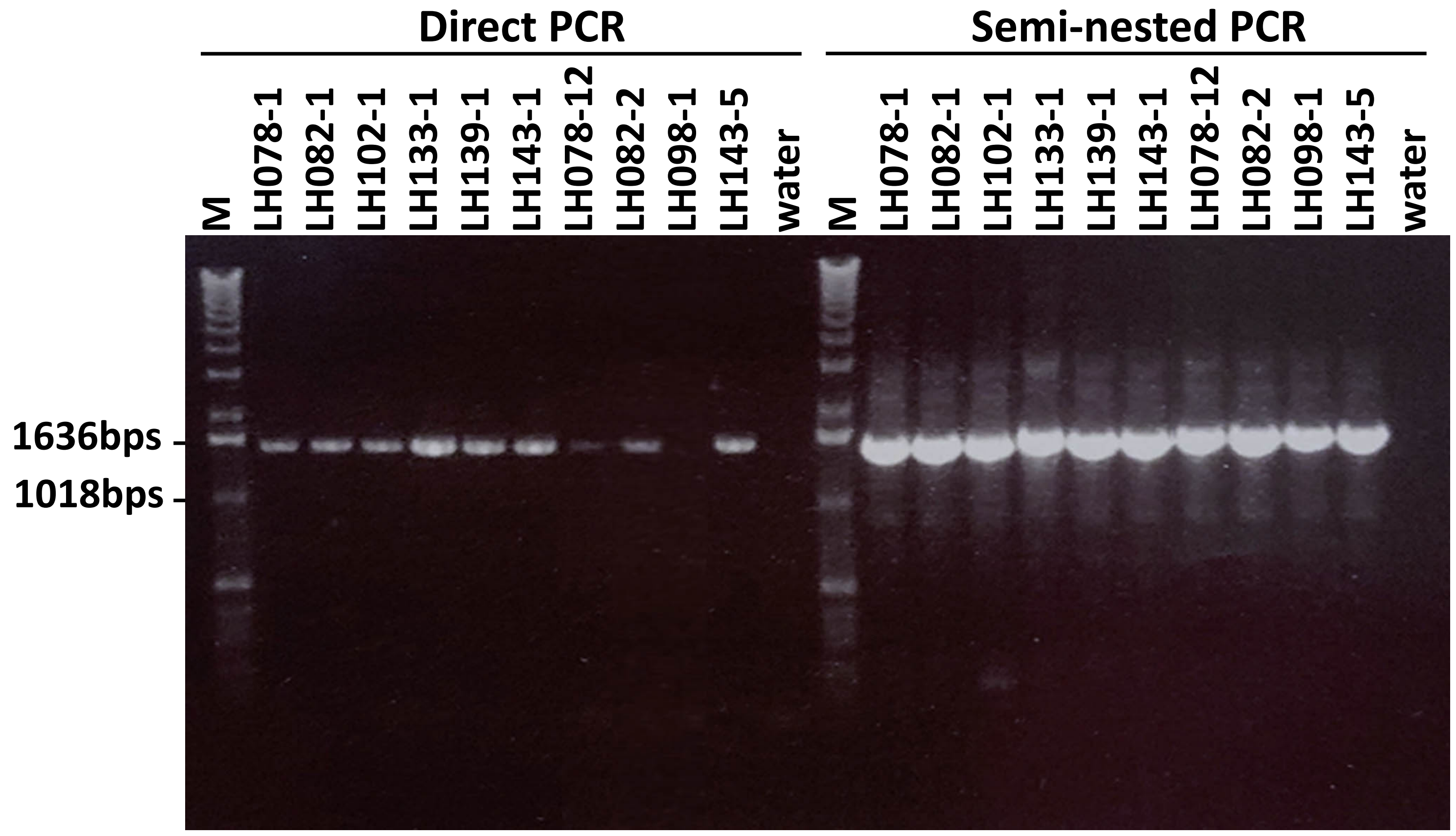

Supplementary Figure 1. Phytoplasma detection in ten leafhoppers by semi-nested polymerase chain reaction (PCR) amplification of 16S rRNA gene with universal primer pair P1/16S-SR (Direct PCR), followed by amplification with primers P1A/16S-SR (Semi-nested PCR). Ten samples include LH078-1, LH82-1, LH102-1, LH133-1, LH139-1, LH143-1, LH078-12, LH082-2, LH098-1, and LH143-5 M: Marker (1Kb DNA ladder); water was used as negative control.

Supplementary Table 1. Detection of phytoplasmas in 76 leafhopper samples by semi-nested polymerase chain reaction (PCR), including 56 quantatitive PCR (qPCR) screend and 20 untested leafhopper samples.

	1-10 sample code/country		11-20 sample code/country		21-30 sample code/country		31-40 sample code/country		41-50 sample code/country		51-56 sample code/country	
**56 (1-56) qPCR screened samples [24]**	LH068-1	USA	LH086-1	Mongolia	LH100-1	Kyrgyzstan	LH116-1	Australia	LH134-1	Australia	**LH078-1***	**South Africa**
	LH070-1	USA	LH087-1	Mongolia	LH103-1	Kyrgyzstan	LH117-1	Australia	LH136-1	Australia	**LH082-1***	**South Africa**
	LH071-1	USA	LH088-1	Mongolia	LH104-1	China	LH118-1	Australia	LH137-1	Australia	**LH0102-1***	**Kyrgyzstan**
	LH072-1	USA	LH089-1	Mongolia	LH108-1	Thailand	LH120-1	Australia	LH138-1	Australia	**LH0133-1***	**Australia**
	LH073-1	USA	LH091-1	Mongolia	LH109-1	Thailand	LH121-1	Australia	LH140-1	China	**LH0139-1***	**Australia**
	LH076-1	South Africa	LH092-1	Republic of Congo	LH110-1	Taiwan	LH126-1	Australia	LH141-1	China	**LH0143-1***	**China**
	LH079-1	South Africa	LH093-1	Republic of Congo	LH111-1	Thailand	LH128-1	Australia	LH147-1	Zambia		
	LH080-1	South Africa	LH095-1	Republic of Congo	LH112-1	Australia	LH130-1	Australia	LH148-1	Zambia		
	LH083-1	South Africa	LH096-1	Kyrgyzstan	LH113-1	Thailand	LH131-1	Australia	LH149-1	Zambia		
	LH085-1	South Africa	**LH098-1**	**Kyrgyzstan**	LH115-1	Taiwan	LH132-1	Australia	LH219-1	Switzerland		

	1-10 sample code/country		11-20 sample code/country	
**20 (1-20) unexamined samples**	LH102-2	Kyrgyzstan	LH078-11	South Africa
	LH078-2	South Africa	**LH078-12**	**South Africa**
	LH078-3	South Africa	LH078-13	South Africa
	LH078-4	South Africa	**LH082-2**	**South Africa**
	LH078-5	South Africa	LH082-3	South Africa
	LH078-6	South Africa	LH143-2	China
	LH078-7	South Africa	LH143-3	China
	LH078-8	South Africa	LH143-4	China
	LH078-9	South Africa	**LH143-5**	**China**
	LH078-10	South Africa	LH133-2	Australia

* represents the leafhopper samples that were detected phytolasma-positive by qPCR [24]. Bolded leafhopper samples (including *) were detected phytoplasma-positive by semi-nested PCR.

[24] Trivellone, V.; Wei, W.; Filippin, L.; Dietrich, C. H. Screening potential insect vectors in a museum biorepository reveals undiscovered diversity of plant pathogens in natural areas. *Authorea.* **2021,** DOI: 10.22541/au.161372577.77393543/v1
